# Effect of Pomalidomide-based regimen on the treatment of patients with first-relapsed multiple myeloma and analysis of prognostic factors

**DOI:** 10.12669/pjms.41.6.10472

**Published:** 2025-06

**Authors:** Qing Fan, Qihang Man, Yu Wang

**Affiliations:** 1 Qing Fan Department of Hematology, Beijing Shunyi Hospital, Beijing 100000, Beijing, China; 2 Qihang Man Department of Hematology, Beijing Astronautics Center hospital, Beijing 100000, Beijing, China; 3 Yu Wang Department of Hematology and Oncology, Beijing Astronautics General hospital, Beijing 100000, Beijing, China

**Keywords:** Pomalidomide, Recurrent refractory multiple myeloma, Progression-free survival, Risk factors

## Abstract

**Objective:**

To explore the effect and safety of pomalidomide (POM)-based regimen on the treatment of patients with first-relapsed multiple myeloma (MM), and to analyze the factors affecting prognosis.

**Methods:**

This was retrospective study. Thirty-eight patients with first-relapsed MM admitted to Hematology Department of Beijing Shunyi Hospital from January 2020 to June 2023 were included and divided into observation group (n=20) and control group (n=18) according to treatment regimens. Record the time of progressive disease (PD), death, adverse reactions, analyze the progression-free survival (PFS) and overall survival (OS).

**Results:**

The overall response rate of the observation group was 85.00%, was higher than that of 55.56% in the control group (c^2^=3.993, *P*=0.046). Seventeen cases and all cases had PD in the observation and the control groups. Two patients in each group died. The OS analysis was not performed as the censoring rate in both groups exceeded 70%. The median PFS of the observation group was 9.118 months, was higher than 4.750 months of the control group (*P*<0.01). Influential factors of PFS were therapeutic regimen-based grouping, risk stratification, LDH>240 U/L, and the presence of extramedullary plasmacytoma or not (all *P*<0.05). The use of POM was protective factor for PFS, extramedullary plasmacytoma, risk stratification, and LDH>240 U/L were independent risk factors.

**Conclusion:**

POM-based regimen can significantly improve the PFS and overall response rate in patients with first-relapsed MM. The use of POM is a protective factor, extramedullary plasmacytoma, risk stratification, and LDH>240 U/L are independent risk factors for PFS.

## INTRODUCTION

Multiple myeloma (MM) is a hematological malignancy characterized by the clonal growth of malignant plasma cells.^1^ The prevalence and incidence rate of MM in China were about 6.88/100,000 and 1.60/100,000, respectively. Its prevalence in females (5.79) was lower than that in males (7.89), and the average age of onset was 57.9 years.^2^ At present, treatment of MM has achieved significant progress, and the overall survival (OS) of patients has been extended than before. However, MM is still incurable, and some patients may progress to recurrent refractory multiple myeloma (RRMM). Available therapeutic options are limited for the treatment of patients with RRMM, posing significant challenges for research on therapeutic drugs and regimens.^3^

The use of conventional cytotoxic drugs and targeted drugs produces unsatisfactory outcome for the treatment of RRMM, and polytherapy may be a better choice. With the research development, and clinical exploration of novel therapeutic agents against MM, as well as the emergence of targeted drug products, combined therapy offers a new strategy for the treatment of RRMM. Pomalidomide (POM) is a third-generation immunomodulatory agent with stronger anti-myeloma activity compared to lenalidomide (LEN). It has been reported that compared with bortezomib + dexamethasone, POM + bortezomib + dexamethasone was more effective and safer in treating RRMM patients, with a longer progression-free survival (PFS).^4^

Moreover, for MM patients with renal insufficiency, POM + dexamethasone could significantly improve the therapeutic effect and overall response rate; and POM + bortezomib + dexamethasone could also significantly improve the median PFS in these patients. In addition, compared with bortezomib + dexamethasone, the addition of POM showed a more significant effect in the treatment of RRMM.^5^ Nevertheless, there is so far few studies on the therapeutic effect and safety of POM since it has only recently entered the Chinese market. This study aimed to observe the clinical efficacy and safety of the pomalidomide containing regimen in the treatment of first-time relapsed refractory multiple myeloma, and to analyze the factors affecting prognosis.

## METHODS

This was a retrospective study. Thirty-eight patients admitted to Beijing Shunyi Hospital from January 2020 to June 2023 were recruited as subjects and divided into two group by treatment method: the observation group (POM-based treatment) and the control group (POM-free treatment), with 20 cases in the observation group and 18 cases in the control group. All patients included in this study had received 1-3 type (s) of treatment in the past, with the first progression during treatment or ineffective response to the last treatment.

### Ethical Approval:

This study was approved by the Institutional Ethics Committee of Beijing Shunyi Hospital (No.: AF/SQ-02/04.0; Date: April 15, 2023), and all patients and their families were informed and consented to participate in this study.

### Inclusion criteria:

- Patients who met the diagnostic criteria of the Guidelines for the diagnosis and management of MM in China (2022 revision).^6^
- Patients over 18 years old with an expected survival time of ≥3 months.
- Patients who had received 1-3 type (s) of treatment in the past, with the first progression during treatment or ineffective response to the last treatment (i.e., disease progression during the applied treatment or within 60 days after completing the treatment).
- Patients who had not undergone allogeneic hematopoietic stem cell transplantation.

### Exclusion criteria:

- Patients diagnosed with non-secretory MM.
- Patients who experienced severe cardiovascular disease within the past six months.
- Patients with severe infectious diseases or communicable disease.
- Patients with deep vein thrombosis or pulmonary embolism within the past year.
- Patients with malignant tumors of other types.
- Patients with mental and neurological disorders who cannot cooperate with treatment.
- Patients who required long-term use of immunosuppressive agents or steroids.

To be specific, the therapeutic regimen in the control group was described as follows: bortezomib (1.3 mg/m^2^/d) through subcutaneous injection on days 1, 8, 15 and 22; and dexamethasone (20 mg/d; or reduced dosage to 10 mg/d for subjects aged >75-year-old) through oral administration or intravenous injection on the day and second day after the application of bortezomib. Every 21 days was a treatment cycle. On the basis of the therapeutic regimen in the control group, patients in the observation group were given 4 mg/d of POM orally (day 1-21). In case of a risk of thromboembolism during treatment, low-molecular-weight heparin or aspirin should be used for prevention as required.

### Outcome measures:

We collected patient information through the hospital medical record management system, including Eastern Cooperative Oncology Group (ECOG) score, age, gender, Durie-Salmon staging (DS), International Staging System (ISS) staging, immune type, serum protein electrophoresis, serum and urine immunofixation electrophoresis, routine blood test, creatinine, lactate dehydrogenase (LDH), β2 microglobulin, albumin, blood calcium, bone marrow cytology, peripheral blood smear at relapse, etc. Through telephone, outpatient, or inpatient follow-up, this study recorded the time of progressive disease (PD), death, and adverse reactions of patients during the treatment process, and analyzed the PFS and OS. The clinical effect was evaluated according to the criteria proposed by the International Cancer Control (UICC) and the World Health Organization (WHO).

The effect was classified into four categories: *Complete response (CR):* complete disappearance of all tumor lesions for at least one month; *Partial response (PR):* shrinkage of the tumor lesion by >50%, no PD for at least one month, and no new lesion in the process; *Stable disease (SD):* shrinkage of the lesion by <50% or lesion increase by <25% for no less than one month; *PD:* tumor lesion increased by >25%, or new lesion (s). Overall response rate = (cases of CR+PR)/total cases×100%. In addition, the safety of treatment was assessed using the National Cancer Institute Common Toxicity Criteria 3.0 (NCICTC3.0).

### Statistical analysis:

All data in this study were statistically analyzed using SPSS20.0 software. Measurement data were expressed as (x¯±s) and enumeration data was expressed as n (%). Corresponding inter-group comparison adopted χ² test and independent sample *t*-test, respectively. Kaplan-Meier curve was constructed to estimate the OS and PFS. Log-rank test was used for univariate analysis of prognostic factors. Influential factors with statistical differences were incorporated in Cox regression model for multivariate analysis. P<0.05 indicates a statistically significant difference.

## RESULTS

No significant difference was observed in the comparison of general data between the two groups of patients at relapse (Table-I). The follow-up deadline was December 31, 2023, there were 16 cases of PD in the observation group, and all cases in the control group.

**Table-I:** Comparison of general data between the two groups.

Items	Observation group (n=20)	Control group (n=18)	t/χ² value	P value
Age	69.25±4.98	69.50±5.90	0. 142	0.888
***Gender***				
Male	11 (55.00%)	10 (55.56%)	0.001	0.973
Female	9 (45.00%)	8 (44.44%)		
***Renal function***				
Normal	12 (60.00%)	9 (50.00%)	0.383	0.536
Insufficiency	8 (40.00%)	9 (50.00%)		
***Lactic dehydrogenase***				
>240U/L	7 (35.00%)	6 (33.33%)	0. 012	0.914
≤240U/L	13 (65.00%)	12 (66.67%)		
***β2 microglobulin***				
≥5.5mg/L	10 (50.00%)	5 (27.78%)	1.958	0.162
<5.5mg/L	10 (50.00%)	13 (72.22%)		
***Albumin***				
>35g/L	13 (65.00%)	16 (88.89%)	2.991	0.084
≤35g/L	7 (35.00%)	2 (11.11%)		
***Serum calcium***				
>2.65mmol/L	3 (15.00%)	5 (27.78%)	0.931	0.335
≤2.65mmol/L	17 (75.00%)	13 (72.22%)		
Extramedullary lesion				
With	4 (20.00%)	5 (27.78%)	0.317	0.573
Without	16 (80.00%)	13 (72.22%)		
ISS staging				
Stage Ⅰ-Ⅱ	8 (40.00%)	8 (44.44%)	0.077	0.782
Stage Ⅲ	12 (60.00%)	10 (55.56%)		
MM classification			0.372	0.985
IgG	10 (50.00%)	8 (44.44%)		
IgA	3 (15.00%)	4 (22.22%)		
IgD	1 (5.00%)	1 (5.56%)		
IgM	1 (5.00%)	1 (5.56%)		
Light-chain type	5 (25.00%)	4 (22.22%)		
Risk stratification			0.001	0.973
High-risk	9 (45.00%)	8 (44.44%)		
Standard risk	11 (55.00%)	10 (55.56%)		

**Fig.1:**
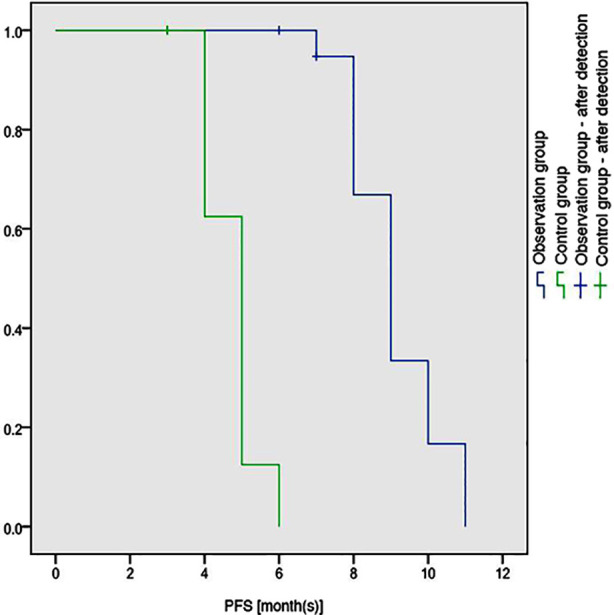
Comparison of PFS curves between the two groups.

The overall response rate of the observation group was significantly higher than that of the control group (85.00% vs. 55.56%; (χ²=3.993, *P*=0.046) (Table-II). The censoring rate of the observation group was 15.00%, and data in this group was used for PFS analysis. While 2 of 20 patients in the observation group died, and 2 of 18 patients in the control group died. The censoring rate of the observation group and the control group was 90.00% and 88.89%, respectively, both of which were above 70%. The median OS had not been reached in either group, which might result in a low accuracy of using the COX model for OS analysis. Hence, OS analysis was not conducted in this study. The median PFS of the observation group was 9.118 months (8.575-9.660), which was higher than 4.750 months (4.415-5.085) of the control group (*P*<0.01; Fig.1), showing obviously prolonged PFS in the former group.

**Table-II:** Comparison of clinical effect between the two groups.

Groups	CR	PR	SD	PD	Overall response rate (%)
Observation group (n=20)	8	9	2	1	17 (85.00)
Control group (n=18)	4	6	4	4	10 (55.56)
*χ²* value					3.993
*P* value					0.046

Log-rank test was further performed on gender, staging, therapeutic regimen-based grouping, risk stratification, LDH, MM classification and other factors of the included 38 patients. The factors affecting PFS included regimen-based grouping (*P*=0.005), risk stratification (*P*=0.005), LDH>240 U/L (*P*=0.014), and the presence of extramedullary plasmacytoma or not (*P*=0.010) (Table-III). Covariates with statistically significant differences (*P*<0.05) in Log-rank test were included for Cox analysis. Data in Table-IV shows that the use of POM was a protective factor for PFS, while extramedullary plasmacytoma, risk stratification, and LDH>240U/L were independent risk factors (Table-IV).

**Table-III:** Log-rank test of influential factors of PFS.

Covariates	Cases	Chi-Square	P	Covariates	Cases	χ² value	P value
Regimen				ISSⅢ stage			
Observation group	20	7.923	0.005	Yes	17	3.164	0.075
Control group	18			No	21		
Gender				LDH>240U/L			
Male	20	0.451	0.502	Yes	17	6.047	0.014
Female	18			No	21		
Risk stratification				β2 microglobulin ≥5.5mg/L			
High-risk	17	7.950	0.005	Yes	16	0.009	0.925
Standard risk	21			No	22		
IgG type				Albumin>35g/L			
Yes	11	0.786	0.375	Yes	16	0.595	0.441
No	27			No	22		
IgA type				Serum calcium >2.65mmol/L			
Yes	15	0.260	0.610	Yes	15	1.222	0.269
No	23			No	23		
Other Ig type				Extramedullary lesion			
Yes	16	0.565	0.452	Yes	19	6.585	0.010
No	22			No	19		
Light-chain type				Renal insufficiency			
Yes	16	0.001	0.982	Yes	16	0.420)	0.517
No	22			No	22		
DSⅢ stage				ECOG score			
Yes	15	0.321	0.571	1-2 point (s)	15	0.367	0.545
No	23			>2 points	23		

**Table-IV:** Cox regression analysis of influential factors of PFS.

Covariates	β	SE	Wald	P	HR	95%CI
Regimen-based grouping	-2.834	1.188	5.693	0.017	0.059	0.006-0.603
Extramedullary lesion or not	2.050	0.898	5.212	0.022	7.772	1.337-45.187
Risk stratification	2.399	0.947	6.420	0.011	11.008	1.722-70.383
LDH>240U/L or not	2.007	0.906	4.907	0.027	7.443	1.260-43.957

In the observation group, grade 3-4 adverse reactions were hematological events and respiratory infections predominantly. In the control group, grade 3-4 adverse reactions were dominated by hematological events in this group. Statistical analysis revealed no significant difference in grade 3-4 adverse reactions between groups (Table-V). Besides, adverse reaction of thrombosis did not cause the death of any patient in both groups.

**Table-V:** Comparison of grade ≥3 adverse reactions between the two groups.

	Observation group (n=20)	Control group (n=18)	P value
***Hematological adverse reactions***			
Granulocytopenia	7 (35.00%)	2 (11.11%)	0.084
Thrombocytopenia	3 (15.00%)	1 (5.56%)	0.344
Anemia	2 (10.00%)	1 (5.56%)	0.612
***Non-hematological adverse reactions***			
Respiratory tract infection	5 (25.00%)	3 (16.67%)	0.529
Gastrointestinal reaction	0	1 (5.56%)	0.285
Thrombosis	2 (10.00%)	0	0.168

## DISCUSSION

The results of this study showed that compared with the bortezomib and dexamethasone regimen, the combination of pomalidomide and bortezomib and dexamethasone regimen significantly improved progression free survival and overall response rate in patients with first-time recurrent multiple myeloma. In this study, the median PFS of the pomalidomide treatment group was 9.118 months, significantly higher than the control group’s 4.750 months, and the efficacy was significant (P<0.01).

The results were consistent with multiple research findings,^7^,^8^ and Cox regression analysis showed that taking pomalidomide was a protective factor for RRMM.^9^ MM is a common hematological malignancy that cannot be completely cured. Although actively treated, it is prone to eventually progress to RRMM.^10^,^11^ Multi drug combination strategy is a better choice for RRMM patients.^12^ Pomalidomide is a third-generation immunomodulatory agent with stronger anti MM activity and safety.^13^ Research has shown that MM patients with extramedullary plasmacytoma have a significantly increased risk of progression compared to those without extramedullary lesions,^14^ indicating that most MM patients have a poor prognosis when extramedullary lesions occur.

The research results show that extramedullary plasmacytoma, genetic risk stratification, and LDH elevation>240 U/L are independent risk factors for PFS, consistent with previous studies.^15^-^20^ No effect of age, hemoglobin level, platelet level, ECOG score, etc. on prognosis was observed. There is currently no specific treatment plan for extramedullary lesions in clinical practice, and there is limited research on this topic. More clinical trials are needed to validate the clinical efficacy of new drugs for extramedullary lesions. The research results show that pomalidomide has activity in adverse genetic risk. These observations provide a theoretical basis for further research on the use of pomalidomide in the treatment of high-risk genetic patients.

However, there are few related studies and more clinical trials are needed to verify its impact on prognosis. Research has shown that age, lactate dehydrogenase levels, hemoglobin and platelet levels, ECOG status, and survival outcomes of MM patients are significantly correlated. MM patients under 65 years old, with normal lactate dehydrogenase levels, hemoglobin ≥10 g/dL and platelets ≥75×10^9^/L, ECOG0-2 status levels, and no extramedullary plasma cell tumor growth have a relatively longer survival period. This may be related to the small sample size and the short follow-up period of this study, which failed to analyze the relationship between OS and influencing factors. A longer follow-up period is needed to obtain more comprehensive results.

The results of this study also showed that multiple complications occurred during the treatment of RRMM patients with pomalidomide related regimens, mainly hematological and respiratory infections. There was no patient discontinuation or death during the treatment process, indicating that RRMM patients have good tolerance to pomalidomide.

### Limitations

This is a retrospective single center study with a small sample size, and the results obtained are limited. It requires the participation of multiple centers and the expansion of the sample size to obtain more comprehensive results. Pomalidomide has a short time on the market in China, and there are few studies on its efficacy and safety. More clinical trials are needed to verify its clinical efficacy and safety, and to analyze the factors that affect patient survival prognosis.

## CONCLUSIONS

This study explored the survival status and prognostic factors of patients with primary recurrent multiple myeloma treated with a combination of pomalidomide, bortezomib, and dexamethasone regimen, and innovatively evaluated the effectiveness of this regimen, which has certain clinical practice guidance. Compared with bortezomib and dexamethasone, the combination of pomalidomide and bortezomib and dexamethasone regimen can significantly improve progression free survival and overall response rate in patients with primary recurrent multiple myeloma. Among them, scheme grouping, risk stratification, LDH>240U/L, and the presence or absence of extramedullary plasma cell carcinoma are factors that affect PFS. Multiple myeloma is difficult to cure, and recurrence after treatment can affect patients’ survival time and increase their economic burden. Pomalidomide provides a new treatment option for patients with recurrent multiple myeloma.

### Authors’ Contributions:

**QF:** Carried out the studies, participated in collecting data, drafted the manuscript, are responsible and accountable for the accuracy or integrity of the work.

**QM** and **YW:** Literature search, Performed the statistical analysis and participated in its design.

All authors read and approved the final manuscript.
